# Trajectories of interbrain synchrony during teamwork: links with team composition and performance

**DOI:** 10.1093/scan/nsaf081

**Published:** 2025-08-05

**Authors:** Coralie Réveillé, Grégoire Vergotte, Gérard Dray, Pierre-Antoine Jean, Stéphane Perrey, Grégoire Bosselut

**Affiliations:** EuroMov Digital Health in Motion, University of Montpellier - IMT Mines d’Alès, Montpellier, France; EuroMov Digital Health in Motion, University of Montpellier - IMT Mines d’Alès, Montpellier, France; EuroMov Digital Health in Motion, University of Montpellier - IMT Mines d’Alès, Montpellier, France; EuroMov Digital Health in Motion, University of Montpellier - IMT Mines d’Alès, Montpellier, France; EuroMov Digital Health in Motion, University of Montpellier - IMT Mines d’Alès, Montpellier, France; EuroMov Digital Health in Motion, University of Montpellier - IMT Mines d’Alès, Montpellier, France

**Keywords:** team dynamics, team cognition, hyperscanning, fNIRS, personality

## Abstract

Teamwork is a dynamic phenomenon that develops over time. Team cognition involved in teamwork is known to increase over successive task episodes. However, there is limited understanding of the trajectory of team cognition within a single task episode. The current functional near infrared spectroscopy hyperscanning study used interbrain synchrony (IBS) to investigate how team cognition develops throughout a task. The links of IBS trajectories with team personality composition and performance were also investigated. Results showed that (i) IBS did not significantly change over time within the sample; (ii) teams show significant differences in IBS trajectories; (iii) team personality did not predict IBS trajectories and; (iv) IBS trajectories did not predict team performance. While IBS was found to appear in our sample, these results warrant replication and additional research is required to better understand IBS trajectories, especially the heterogeneity across teams.

## Introduction

Individuals rarely act alone in their daily lives; instead, collaborative work is ubiquitous, from the workplace to leisure activities like team sports. The success of these collaborative efforts relies not only on individual skills but, more importantly, on the quality of interactions among team members (LePine et al. 2008, Schmutz et al. 2019). In critical contexts, like surgical teams or flight crews, ineffective collaboration or difficulty in establishing teamwork can have disastrous consequences (Salas et al. 1999). This makes it crucial to understand the dynamics of teamwork that operate during a task execution.

Teamwork is defined as a dynamic process in which two (or more) interdependent individuals make collaborative efforts to perform coordinated behaviours in order to reach a common goal (Salas et al. 1992, McEwan and Beauchamp 2014). During teamwork, various team-level phenomena—referred to as emergent states—arise (e.g. team cohesion, team trust, team cognition, Kozlowski and Bell 2003). They develop progressively as teamwork unfolds, making teamwork a dynamic phenomenon (Arrow et al. 2000, Luciano et al. 2018). Emergent states may follow two developmental dynamics (Kozlowski et al. 1999): the first—divergent—leads to a differentiation between team members while the second—convergent—leads to increased similarity between them (Kozlowski et al. 1999).

Decades of research provided significant insights on emergent states trajectories (Fyhn et al. 2023), i.e. the rate of change in the emergent states over time (Larson et al. 2020). Team cognition—the way in which knowledge is represented and/or distributed among team members (Niler et al. 2021) follows a convergent trajectory, meaning that the shared knowledge among individuals tend to increase over time (e.g. Marks et al. 2001, Kneisel 2020). Additionally, team cognition develops earlier and faster than other emergent states (e.g. team cohesion, team confidence, Fyhn et al. 2023), and its trajectory is an important predictor of team performance (Niler et al. 2021, Fyhn et al. 2023).

However, the methodological approaches used in these studies leave gaps in our understanding of team cognition development (Kozlowski and Chao 2012, Mathieu et al. 2017). Most existing research relies on retrospective questionnaires administered after task completion, capturing only static snapshots of successive task episodes. This approach does not reveal how emergent states evolve within a task episode (Fyhn et al. 2023). To gain deeper insight into these trajectories, continuous and unobtrusive measurements are needed (Delice et al. 2019; Klonek et al. 2019). Sensor-based technologies (e.g. Rosen et al. 2015, Kolbe and Boos 2019) including neurophysiological measurements, offer a way to collect objective data throughout task execution (Luciano et al. 2018, Ramos-Villagrasa et al. 2018, Shuffler et al. 2020).

Interbrain synchrony (IBS) has been proposed as a way to assess team emergent state (Wang et al. 2021, Lu et al. 2022, Réveillé et al. 2024). IBS is defined as the level of similarity in brain activity between interacting individuals (Czeszumski et al. 2020), and can be measured using hyperscanning methods that simultaneously record the brain activity of interacting individuals (Montague et al. 2002). IBS quantifies the strength of the brain-to-brain coupling between individuals (Hakim et al. 2023, De Felice et al. 2024), and allows to uncover the neural underpinnings of cognitive phenomenon unfolding during social interaction (Misaki et al. 2021). A meta-analysis (Czeszumski et al. 2020) demonstrated that significant IBS arises when individuals cooperate together towards a common goal, as compared to other conditions (e.g. rest, parallel game, competition). More precisely, IBS appears in prefrontal cortex (PFC) and temporal-parietal junction (TPJ), highlighting the importance of brain regions involved in social cognition (e.g. mentalizing system and mirror neuron systems) when individuals work together (Babiloni and Astolfi 2014, Balconi et al. 2018). Another recent meta-analysis, established a connection between IBS and the theoretical model of teamwork (Réveillé et al. 2024). The results show that: (i) IBS is affected by team characteristics such as team composition (defined as the configuration of member attributes in a team, Bell 2007); (ii) IBS in turn influences team performance (Réveillé et al. 2024); and (iii) IBS is associated with various emergent states measured by questionnaires (e.g. feeling of cooperativeness). All this evidence seems to suggest that IBS may reflect a team-level phenomenon. Some authors extended this idea further, suggesting—explicitly or implicitly—that IBS reflects certain aspects of team cognition (Stevens et al. 2010, Filho et al. 2016).

The few studies examining the trajectory of IBS during a task episode have yielded contradictory findings. Some have reported a significant increase in IBS within the PFC and TPJ during brainstorming tasks, where participants took turns generating words (approximately 5 min), or during tasks involving managing a healthcare crisis during less than 20 minutes (Lu and Hao 2019, Xu et al. 2019). Other studies observed a decrease in IBS within the PFC and TPJ when participants performed coordinated actions, such as collaboratively assembling small objects (10 minutes) or jointly driving a car in a video game during 15 minutes (Mayseless et al. 2019, Wikstrom et al. 2022). Finally, several studies found no significant changes in IBS when participants alternately generated words orally over short durations of 8 minutes or less (Wang et al. 2019, Lu et al. 2020, Duan et al. 2022). These inconsistencies highlight the need for further investigation using longer task durations, allowing sufficient time for IBS to develop.

In addition to understanding its trajectory, it is also crucial to examine the factors that shape it, and its potential consequences for teamwork. Since the level of IBS during a task is linked to team characteristics and performance (Réveillé et al. 2024), the development of emergent states is similarly influenced by team personality composition (Bell et al. 2018, Larson et al. 2020) and predicts performance (Fyhn et al. 2023). More specifically, teams with higher levels of agreeableness (i.e. the tendency to be friendly and compassionate, Goldberg 1990) and conscientiousness (i.e. the tendency to be organized and self-disciplined, Goldberg 1990) tend to show greater increment in emergent states (e.g. team cohesion, conflict management) throughout multiple task episodes (Acton et al. 2020, Larson et al. 2020).

To address the gaps in the literature regarding the development of team emergent states during a single task episode, the present study builds on the assumption that IBS may serve as a measure of team cognition and aims to investigate its trajectory. Additionally, it examines the influence of team characteristics, and the effect on team performance. Based on the existing literature, we propose the following hypotheses.

*Hypothesis 1:* IBS in the PFC and TPJ will increase across the duration of a teamwork task.

*Hypothesis 2:* Team personality composition will influence the IBS development, with higher levels of team conscientiousness and agreeableness predicting greater increases in IBS.

*Hypothesis 3:* Greater increases in IBS will influence a better team performance.

## Materials and Methods

The current study’s protocol was pre-registered (https://osf.io/xgj4e/).

### Participants

98 participants were recruited. Inclusion criteria were healthy adults, with French as native language, with no neurological history. There was no restriction on handedness. Two-persons teams (dyads) were composed of unacquainted participants, with no specification of gender composition (i.e. mixed or same gender). As 10 participants provided unusable data (see section ‘Pre-Processing and Visual Quality Check’), 9 dyads were excluded from the analysis. The final sample is thus composed of 40 dyads (4 female-female, 14 male-male, 6 female-male, age = 22.5 ± 3.0 years) in agreement with the expected number of dyads calculated with a priori analyses (*N*_expected_ = 35). Descriptive statistics of the final sample are presented in Supplementary Material 1.

### Experimental task

The experiment conducted in an insulated lab room was approved by the local Research Ethics Committee (UM 2024-007bis). The experiment involves a computer-based task where participants must cooperatively reproduce a path. This task is inspired by the MapTasks corpus (Anderson et al. 1991) and has previously been employed in hyperscanning studies (Stevens and Galloway 2014, Réveillé et al. 2022).

**Figure 1.**
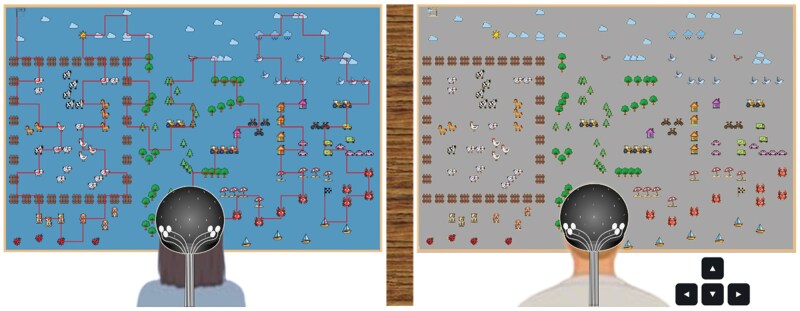
Experimental task: a dyad before the start of the experimental task. On the left: the Guide in front of the screen on which appears the path to be reproduced. In the middle: the curtain separating the two participants. On the right: the Drawer, looking at his screen and able to move the cursor, initially placed on the start point (top left) using the arrows on the keyboard.

For each dyad, participants were randomly assigned the roles of Guide and Drawer before the start of the experiment. Participants were seated side by side, each facing a screen (61 × 36 cm, width × height), positioned 60 cm apart, and separated by a curtain to prevent them from seeing each other’s screen. Before the beginning of the task, both Guide and Drawer screens displayed a map with start and end points, and strictly identical icons. At the beginning of the task, the Guide’s screen also displayed a path going from start to end, and passing through icons, called the ‘Reference Path’. On the Drawer’s screen, this path was missing, but a cursor was placed on the starting point (Fig. 1). When the ‘Enter’ key on the Drawer’s keyboard was pressed, the cursor moved straight across the screen at a constant and low speed (5.70 s/cm), drawing a path on its way. The cursor could not be slowed down nor sped up, but could be redirected at any time by pressing one of the four arrows on the keyboard.

Dyads were informed that they were a team working towards a common goal. They had to work together so that at the end of the task, the same on the Drawer’s screen was exactly the same as the one already on the Guide’s screen. Participants were specified (i) they were free to verbally communicate; (ii) the tasks would end after 30 minutes; and (iii) their only goal was the spatial accuracy of the path to be drawn. Dyads first performed a 1-minute familiarization task, then a trial (30 minutes), with 3 min rest before and after the task. The detailed instruction given to the participants can be found in Supplementary Material 2.

### Interbrain synchrony trajectories: measure and analysis

#### Brain activity measurement and processing

**Figure 2.**
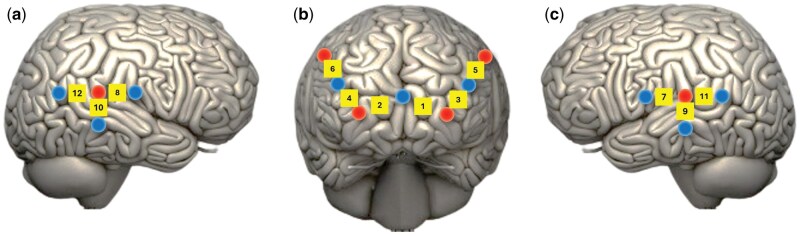
Visualization of the 12 fNIRS channels. (a) Right hemisphere: right TPJ; (b) Front view: left and right fpPFC; (c) Left hemisphere: left TPJ. Circles: sources or detectors; squares: channels. fp = frontopolar; dl = dorsolateral; PFC = prefrontal cortex; TPJ = temporoparietal junction.

Functional near infrared spectroscopy (fNIRS) method was used to measure brain activity in specific brain areas with the NIRScout platform associated with NIRStar software version 15.3 (NIRx GmbH, Berlin, Germany). For each participant, 6 sources and 9 detectors were positioned 30 mm apart (Reindl et al. 2019) forming 12 channels (CH) and covering six regions of interest (ROI): ROI1= left fpPFC (CH 1); ROI2 = right fpPFC (CH 2); ROI3 = left dlPFC (CH 3, 5); ROI4 = right dlPFC (CH 4, 6); ROI5 = left TPJ (CH 7, 9, 11); ROI6 = right TPJ (CH 8, 10, 12) (see Fig. 2). The sampling rate was 7.81 Hz and the fNIRS system used LED sources with two wavelengths, 760 nm targeting deoxyhaemoglobin (HHb) and 850 nm targeting oxyhaemoglobin (O_2_Hb). Lab Recorder from the lab streaming layer was used for data synchronization between fNIRS data and task triggers.

The fNIRS data processing was based on Nguyen et al. (2021) and Reindl et al. (2019) recommendations, and performed using Homer toolbox for Matlab (Huppert et al. 2009, Homer2 package, Matlab R 2021b). The pipeline was as follows: (i) Raw intensity data were converted to Optical Density (OD); (ii) Head movement artefacts were corrected by combining spline interpolation method (p spline = 0.99; Scholkmann et al. 2013), followed by wavelet based signal decomposition (t Motion = 0.5, t Mask = 3.0, STD threshold = 20.0, and AMP threshold = 5.0; Molavi and Dumont 2012); (iii) To mitigate the influence of systemic physiological artifacts in the fNIRS data, a principal component analysis (PCA) was applied, preserving components accounting for 80% of the total variance (Zhang et al. 2005, Bonilauri et al. 2021, Hu et al. 2021); and (iv) Corrected OD signals were converted to O_2_Hb and HHb concentration data following the modified Beer-Lambert law. The power spectral density of each O_2_Hb signal was then visually examined, to check the presence of heartbeat peak around 1 Hz. 23% of the channels did not present the expected peak and were considered bad and excluded. The remaining good channels were averaged inside each of the six ROIs. ROIs were excluded if all their channels were considered bad. Participants who had more than one missing ROI were excluded from analysis. The final sample consists of 40 dyads, four of which have missing data in one ROI (3 in the right dlPFC and one in the left dlPFC; see the flow chart of data selection in Supplementary Material 3).

#### IBS quantification and IBS trajectory extraction

**Figure 3.**
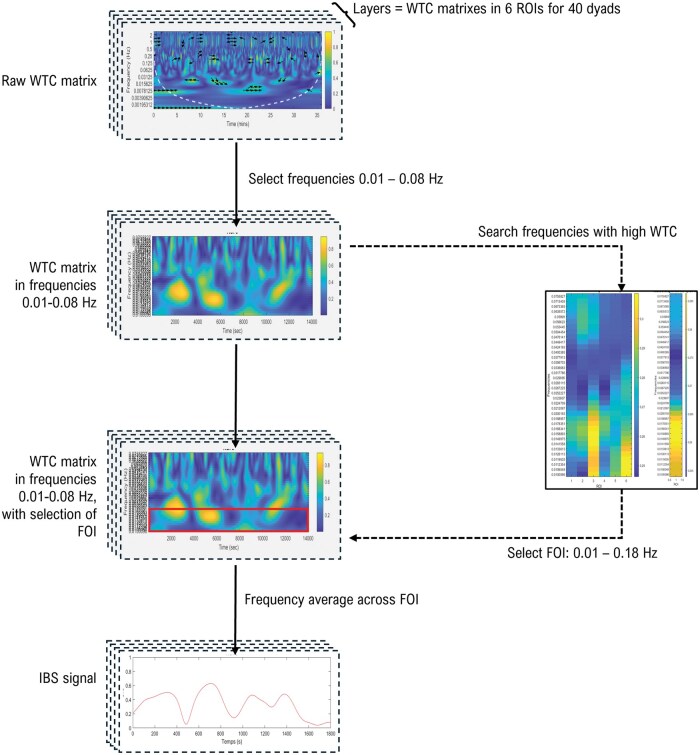
Extraction of IBS trajectory from WTC matrix.

IBS quantification was conducted using Matlab software. Considering the better sensitivity to cortical activation and brain synchrony (Strangman et al. 2002, Xu et al. 2019), O_2_Hb signals were used to quantify IBS (analyses with HHb are available in Supplementary Material 6). Homotopic IBS (i.e. synchrony between the same ROIs) was calculated using Wavelet Transform Coherence (WTC) following the recommendations of Reindl et al. (2019), using ‘wcoherence’ Matlab function. WTC, the most frequently used analysis in fNIRS hyperscanning (Balters et al. 2020, Hakim et al. 2023), is conceptualized as a localized correlation coefficient between two signals in the time and frequency domains [for further explanation, see Chang and Glover (2010) and Grinsted et al. (2004)]. Magnitude-squared wavelet coherence values (‘wcoh’) of the WTC matrix corresponding to the cone of influence (‘coi’) were excluded to avoid estimation bias due to edge effects (Grinsted et al. 2004). Only values corresponding to cortical frequencies (i.e. [0.01; 0.08] Hz) were conserved in order to remove physiological noise (i.e. cardiac frequencies ∼1 Hz, Mayers waves ∼0.1 Hz, and respiration ∼0.2–0.3 Hz, Molavi and Dumont 2012). IBS trajectory was examined in the frequency band of interest (FOI) showing highest IBS. As proposed in hyperscanning guidelines, FOI was identified through visual inspection (Reindl et al. 2019). To minimize the potential bias from visually analysing of 416 WTC matrixes, coherence values were averaged across time (0–30 minutes) and dyads, reducing the data to six WTC vectors corresponding to the six ROIs. This process led to the selection of the frequency band between 0.010 and 0.018 Hz. Finally, to obtain a signal of IBS over the task episode, coherence values were averaged across this FOI (Fig. 3).

### Team personality and performance

Prior to participate to the experiment, participants completed the French version of the Big Five Inventory (Lignier et al. 2023). In this 60-item questionnaire, they had to answer on a five-point Likert scale ranging from 1 (‘disagree strongly’) to 5 (‘agree strongly’). By averaging the answers across the 12 items of each trait, this questionnaire allows to individual personality scores to be calculated in relation to the five personality traits (i.e. Agreeableness (A), Extraversion (E), Conscientiousness (C), Openness to experience (O), and Neuroticism (N)). Cronbach’s α showed acceptable to good internal consistency in the five dimensions of the questionnaire (αA = 0.71; αE = 0.86; αC = 0.84; αO = 0.85; αN = 0.88). Team personality composition was then quantified as the average of scores of the two dyad members (Chan 1998, Bell et al. 2018).

**Figure 4.**
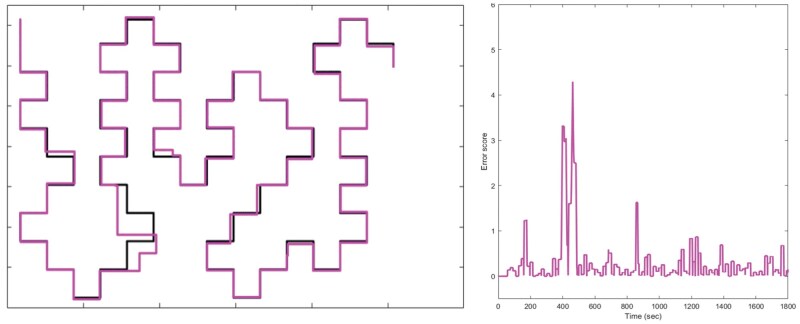
Example of realization of team performance in one dyad. Left: The Reference path, and the path built by the dyad; Right: Local errors as a function of time. Error score = 4400.

Team performance was estimated as the accuracy of the path drawn (i.e. the distance between the path drawn by the dyad and the reference path to be copied). To obtain this distance, the coordinates of the moving cursor were recorded during the task at 10 Hz. For each point of the dyad’s path, a local error value was calculated as the Euclidean distance between the current point and the Reference path. When a point is exactly on the path, the local error is zero, the further away a point is, the higher the local error value. The overall task performance score corresponds to the sum of the local error values (see Fig. 4).

### Statistical analyses

Statistical analyses were conducted using R software, version 4.2.2. (R Core Team 2023).

#### Control analyses

Control analyses were conducted to ensure that the IBS observed in dyads was not spurious. IBS was computed among homotopic brain regions in virtual dyads, which were defined as all possible pairings of one Guide and one Drawer who did not actually interact with one another (Reindl et al. 2019). IBS in real dyads was compared to IBS in virtual dyads using Mann-Whitney tests to deal with the non-normality of the data.

#### IBS trajectory

To test the first hypothesis regarding IBS trajectories, linear mixed models—one per ROI—were fitted using packages {lme4} (Bates et al. 2015) and {lmeTest} (Kuznetsova et al. 2020).

To estimate the trajectory of IBS, we used the overall IBS signal across the task episode (see section ‘IBS trajectory extraction’). First, the overall IBS trajectory of the 40 dyads was estimated using full models (Full Models) including IBS as the response variable, Time as a fixed effect, and slope and intercept as random effects for the dyads (IBS ∼ Time + (1 + Time | ID)). Model fit was estimated using lme4::lmer function with restricted maximum likelihood ratio. The significance of the overall fixed effect of time (i.e. is the IBS trajectory significantly increasing or decreasing?), was tested using the lmeTest::lmer function on the Full Models. Second, to objectively test for the presence of significant inter-dyad differences in IBS trajectories (i.e. slopes), the above-mentioned Full Models were compared to their corresponding reduced models (Reduced Models) that did not include a random slope, but only a random intercept for dyads (IBS ∼ Time + (1| ID)), with an ANOVA using the maximum likelihood ratio test.

#### Team personality composition and team performance

The random effect of time (i.e. the value of the IBS slopes for each dyad) was then extracted. Linear regression models were used to test the effect of the five team personality traits and the effect of IBS trajectories on team performance. *P*-values were corrected with FDR corrections (Benjamini and Yekutieli 2001) to avoid type I errors.

## Results

### Control analyses

Results of the control analyses are presented in Table 1. In four out of the six ROIs—specifically the left and right dorsolateral PFC and the left and right TPJ—IBS was significantly higher in real dyads compared to permuted dyads. This suggests that IBS observed in real dyads was not spurious. Therefore, subsequent analyses focused on the four ROIs where significant effects were observed.

**Table 1: Results of the control analyses (Mann-Withney U tests) for the frequency band of interest (0.01-0.018 Hz):** 

ROI	IBS in real dyads	IBS in permuted dyads	*t*	*P*	Cohen’s *d*
	Mean (SD)	Mean (SD)			
Left fp PFC	0.27 (0.06)	0.26 (0.05)	0.807	.21	0.129
Right fp PFC	0.27 (0.05)	0.27 (0.05)	0.708	.24	0.113
Left dl PFC	0.30 (0.07)	0.25 (0.07)	4.435	<.001	0.719
Right dl PFC	0.27 (0.06)	0.24 (0.09)	2.287	.011	0.381
Left TPJ	0.28 (0.07)	0.26 (0.05)	2.631	.004	0.421
Right TPJ	0.29 (0.07)	0.26 (0.05)	3.923	<.001	0.628

*Note:* fp = frontopolar; dl = dorsolateral; PFC = prefrontal cortex; TPJ = temporoparietal junction; SD = standard deviation.

### IBS trajectories

A visualization of IBS trajectories and the random slope for each dyad is available in Supplementary Material 4. First, regarding the question of IBS increase over time, tests of the fixed effects (i.e. Intercept and Time) of the full models showed that in the four ROIs, the effect of Time on IBS was not significantly different from zero (Table 2).

**Table 2: Results of linear mixed models with random effect of time in the four ROIs (Full Models).** 

		Intercept			Slope		
ROI	*N*	Value	*t*	*P*	Value	*t*	*P*
Left dl PFC	39	0.32	16.63	<.001	−2.71 × 10^−5^	−1.24	.22
Right dl PFC	37	0.27	17.34	<.001	2.14 × 10^−6^	0.10	.92
Left TPJ	40	0.29	19.62	<.001	−1.65 × 10^−5^	−1.02	.32
Right TPJ	40	0.29	16.97	<.001	8.47 × 10^−6^	0.59	.56

*Note:* dl = dorsolateral; PFC = prefrontal cortex; TPJ = temporoparietal junction.

Second, regarding the presence of inter-dyad differences in IBS trajectories (i.e. random effect of Time), comparisons between the full and the reduced models (respectively with and without random effect of slope) showed a lower Akaike Information Criterion for the Full Models compared to the Reduced Models, and a significant difference of the ANOVAs results in the four ROIs (Table 3), showing the existence of significant inter-dyad differences in IBS trajectory in the four ROIs.

**Table 3: Results of the model comparisons in the four ROIs (Models 1 vs. Models 2):** 

	Model 1 (No random slope)			Model 2 (Random slope)			ANOVA		
ROI	AIC	BIC	logLik	AIC	BIC	logLik	Chi²	df	*P*
Left dl PFC	−100975	−100936	50491	−115077	−115019	57544	14106	2	<.001
Right dl PFC	−102233	−102195	51121	−113847	−113789	56929	11617	2	<.001
Left TPJ	−108686	−108647	54347	−121968	−121911	60990	13286	2	<.001
Right TPJ	−104080	−104041	52044	−118364	−118306	59188	14289	2	<.001

*Note:* dl = dorsolateral; PFC = prefrontal cortex; TPJ = temporoparietal junction; AIC = Akaike Information Criterion; BIC = Bayesian Information Criterion; logLik = log-likelihood; df = degree of freedom.

### Relationship with team personality composition and performance

Linear regressions showed that team Openness to experiences predicted IBS slope in one ROI (i.e. Left TPJ: β  =  7.56 × 10^−5^; *P* = .02). However, the significance did not survive FDR corrections. IBS trajectories did not predict team performance. Results are presented in Supplementary Material 5. A sensitivity power analysis was conducted using G*Power (v3.1) for a simple linear regression, with α set at 0.05 and power (1 − β) at 0.80. The results indicated that, given our sample size, the smallest detectable effect size was *f*^2^ = 0.206, corresponding to *R*^2^ ≈ 0.17. The observed effect sizes in our regressions were all below *R*^2^ = 0.13, suggesting that the study lacked sufficient power to detect effects of small to moderate magnitude. As a result, the absence of statistically significant findings may reflect limited sensitivity rather than a true absence of an effect.

### Exploratory analyses

As an exploratory analysis, we investigated whether the average level of IBS during the task predicted team performance using linear regression models. The results did not reveal any significant effects (see Supplementary Material 7 for detailed results).

## Discussion

The current study investigated the trajectory of IBS during a single task episode, and its links with team personality composition and performance.

Based on the assumption that IBS could be a measure of team cognition (Stevens et al. 2010, Filho et al. 2016), we hypothesized a progressive increase in IBS throughout a task episode. However, our findings did not support this hypothesis. This suggests that while subjective measures indicate a perceived growth of team cognition, this progression may not be mirrored by objective continuous IBS measures. As supposed that IBS is a measure of team cognition (Stevens et al. 2010, Filho et al. 2016), a possible explanation for these differing results is that self-reported team cognition questionnaires and IBS may capture different facets of the construct. Indeed, team cognition is a multifaceted concept that encompasses various team-level phenomena, including shared knowledge, mental models, collective memory, and information exchange (Mohammed et al. 2000, Salas et al. 2008, Cooke et al. 2013, Niler et al. 2021). Notably, previous studies investigated team cognition over multiple tasks episodes distributed over extended periods—days, weeks, or months—while the current study focuses on its development within the relatively brief timespan of a 30-minute task episode. This raises the question of whether different components of team cognition unfold over distinct timescales and follow unique developmental trajectories.

As the neurophysiological markers take source in participants’ behaviours (e.g. Müller et al. 2018, Hamilton 2021, Hakim et al. 2023, Koul et al. 2023), a global increase in IBS over the course of the task could have reflected the intuitive notion that participants gradually became more familiar with each other and the task, resulting in smoother interactive behaviours. The current results show no increase in IBS, which may be due to the consistent behaviours of the participants throughout the task. Participants were assigned to fixed roles, with the Guide providing verbal instructions (occasionally questioning the Drawer) and the Drawer responding by pressing keyboard keys (occasionally offering feedback or requesting clarification). In addition, the absence of a significant trend in IBS levels observed in this study should not be taken as evidence of a complete absence of change in IBS. Rather than following a simple monotonic increase or decrease, IBS may instead exhibit more complex temporal patterns, such as cyclic dynamics reflecting the recursive nature of participants’ interactive behaviours (Marks et al. 2001, Luciano et al. 2018). In this context, advanced time series analyses—such as cross recurrence quantification analysis (Marwan et al. 2007)—for detecting potential recursive temporal structures within IBS signals (e.g. Gorman et al. 2012, Mønster et al. 2016). The high temporal resolution of IBS measurements provides a valuable opportunity for future research to investigate these cyclic dynamics.

As linear mixed models confirmed the existence of significant variations in IBS trajectories across teams, we sought to identify the factors underlying the observed inter-team differences in IBS trajectories. Based on previous research, we investigated team personality composition and team performance as potential explanation. After FDR correction, neither regression analyses nor cluster based complementary analyses found links between IBS trajectories, team personality and performance. This contrasts with previous findings suggesting that the development of team level phenomena across multiple task episodes is influenced by team personality—particularly agreeableness and conscientiousness (Acton et al. 2020, Larson et al. 2020), as well as by team performance (Fyhn et al. 2023). In addition to the limited statistical power of this study, two key factors may explain these differences. First, the time span of the current study’s task (30 minutes) may have been too short for team personality to meaningfully influence IBS or for IBS to impact performance. Indeed, team composition shapes emergent states through a complex, time-dependent process (Bell 2007) and early-stage team cognitive phenomena tend to have weaker effects on performance than those developing later (Mohammed et al. 2015). If these relationships take time to emerge, future research should explore the duration required for team personality to affect IBS and for IBS to influence performance. The second point refers to the study setting (Moffat et al. 2023): personality expression is shaped by situational factors (McCrae and Costa 1999, Robinson 2009), and prior research suggests that team personality effects are stronger in laboratory settings than in real-world organizations (Bell 2007). Similarly, the relationship between team level phenomena and performance appears weaker in lab environments compared to real-world contexts (DeChurch and Mesmer-Magnus 2010) which may also contribute to the absence of significant findings.

While personality does not account for inter-team differences in IBS trajectories, other team-level characteristics may be a contributing factor to these variations. Research has shown that various team characteristics influence IBS (for a systematic review and meta-analysis, see Réveillé et al. 2024). Although we have controlled for participants’ familiarity with each other (unacquainted) and with the task (previously unknown to participants), differences between teams on other variables—such as age, team gender composition, or differences in status—could contribute to the observed inter-team differences in IBS. Finally, the observed inter-team differences in IBS trajectories may also be explained by the diversity in individual behaviours. The task used in this study was unscripted and longer compared to other studies examining the evolution of IBS over time. This allowed for a wide range of behaviours, communication styles, problem-solving strategies, and levels of mutual understanding to emerge. Since IBS originates from individuals’ behaviours (Hamilton 2021), future studies should explore the relationship between verbal and motor behaviours within teams and IBS trajectories.

Taking together, the results of the present study do not show a clear development of IBS over the course of a task episode, nor does it establish a link between IBS trajectories, personality, or performance. These findings raise important questions regarding the validity of IBS as a marker of team cognition. While IBS observed during teamwork may indeed reflect a team-level phenomenon, it may not specifically capture the psychological construct referred as team cognition. To determine whether the idea that IBS measures team cognition is valid, it is essential to clarify the meaning of IBS. To this end, further research is needed to explore the relationship between IBS and subjective measures of team cognition, as well as to investigate the temporal dynamics of IBS. Such studies are crucial in identifying which aspects of teamwork IBS actually reflects (Réveillé et al. 2024). Until then, caution is warranted when interpreting the psychosocial significance of IBS (Gvirts Provolovski and Perlmutter 2021; Hamilton 2021; Holroyd 2022).

The absence of significant results may also be linked to methodological aspects, notably the method used to quantify IBS. In this study, IBS was measured using WTC in homotopic brain regions, following established fNIRS hyperscanning approaches (Nguyen et al. 2021, Reindl et al. 2022). However, given the clear role differentiation between participants and the resulting behavioural asymmetry, alternative methodological approaches could have been more suitable. For example, computing IBS across all possible ROI pairings—which encompassing 16 combinations (4 × 4 ROIs)—could offer a more comprehensive approach (Misaki et al. 2021). Similarly, in interactions similar to leader-follower dynamics, implementing time-lagged WTC could help account for temporal delays between the Guide’s actions and the Drawer’s responses. However, determining the optimal time window for incorporating such delays remains an open methodological challenge. Additionally, directional connectivity measures, such as Granger causality, could yield a more refined representation of the directional influence of the Guide’s verbal instructions on the Drawer’s subsequent actions (Redcay and Schilbach 2019; Hamilton 2021).

Finally, this study does confirm that IBS emerges in brain areas associated with social cognition notably dorsolateral PFC and TPJ (e.g. Saxe and Kanwisher 2003, Amodio and Frith 2006, Czeszumski et al. 2020). It also expands understanding of how IBS occurs during teamwork. Control analyses showed that the IBS observed in real teams is not spurious or merely related to the task itself but rather due to the collaborative nature of the interaction. This finding, initially intended as a validation step prior to further analyses, also highlights that IBS can arise during interactions where individuals engaged in distinct and complementary roles. Studies examining IBS in asymmetric social interactions remain scarce (e.g. Toppi et al. 2016, Dehais et al. 2021). The field is largely dominated by research using tasks where participants perform identical actions [e.g. tapping fingers, handling objects, or listing ideas, as seen in studies by Dai et al. (2018), Fishburn et al. (2018), K. Lu et al. (2020)]. As real-world teams are typically composed of individuals with complementary roles (Hasson and Frith 2016), it is important to emphasize that IBS also occurs between individuals fulfilling interdependent and distinct roles.

The current study has limitations. First, based on the available literature, IBS was studied in brain regions linked to social cognition, including the PFC and the TPJ, which are consistently associated with teamwork (Czeszumski et al. 2020). However, it is still possible that IBS emerges and changes over time in other brain regions. Second, although visual inspection is a commonly used approach to select FOI, it has notable limitations, including subjectivity and observer bias. Alternative methods, such as statistical approaches (e.g. one-sample *t*-tests comparing task versus rest, Nozawa et al. 2016) or data-driven techniques (e.g. clustering or machine learning), also carry potential risks. Using the same dataset both to select the BOI and to estimate effects, induces a methodological bias known as double dipping (Kriegeskorte et al. 2009). This circular reasoning (Vul and Kanwisher 2010) compromises statistical independence and can lead to an artificial overestimation of effect sizes. Using separate datasets—one for selecting FOI and another for testing effects—appears to be a promising solution. More broadly, it is essential for the fNIRS community to adopt more transparent practices, such as predefining—and ideally preregistering—the FOI, and to draw inspiration from the efforts made in the fMRI field to promote more robust statistical practices. A third limitation is the absence of short-distance measurement, which could have helped to isolate and remove systemic parameters or to implement a systemic physiology enhanced fNIRS approach. This may have affected the accuracy of IBS measurements, as global physiological fluctuations were not adequately accounted for. Future studies could either incorporate short-range channels to better control for systemic parameter changes, as recommended in Yücel et al. (2021), or assess the components of the low frequency oscillations signal influence by correlating each fNIRS signal with the participant’s own concurrent peripheral data (i.e. Δ[O2Hb] of the fingertip, Li et al. 2018). Forth, despite the fact that coupling in brain activity arises from the transfer of social information conveyed through individuals’ behaviours (Hamilton 2021, Hakim et al. 2023, TenHouten et al. 2023), we only recorded participants’ cortical activity. Investigating teamwork behavioural processes, such as team communication (Cooke et al. 2003), or body movement synchrony (Richardson et al. 2007, 2014) may provide further insights into development of teamwork emergent states, as well as an explanation for inter-team differences in IBS trajectories. Ultimately, team personality composition was operationalized using the mean of individual scores, in line with previous recommendations (e.g. Bell 2007, Larson et al. 2020). However, given the lack of consensus, alternative aggregation methods such as computing the variance of individual scores, could also have been employed (e.g. Vangrieken et al. 2017).

In conclusion, this study aimed to examine how team cognition develops over the course of a single task of 30-minute duration. While we observed no overall increase in IBS trajectories across the sample, we identified significant variations in IBS across teams, meaning that teams may exhibit unique interaction patterns. Future research could benefit from a multisource approach that integrates both subjective reports and physiological measures, in order to deepen our understanding of the functional significance of IBS in teamwork and to clarify its potential links with team cognition.

## Supplementary Material

nsaf081_Supplementary_Data

## Data Availability

The datasets generated and analysed during the current study are available from the corresponding author on reasonable request.
