# Supplementary material for: Trajectories of interbrain synchrony during teamwork: links with team composition and performance
Source: Soc Cogn Affect Neurosci. 2025 Aug 5;20(1):nsaf081. doi: 10.1093/scan/nsaf081 (PMC12548022; doi:10.1093/scan/nsaf081)
Supplement: nsaf081_Supplementary_Data [file nsaf081_supplementary_data.docx]

# Supplementary material

## Supplementary material 1. Description of the sample

**Table SM-1:** description of the sample: age and gender (for individual participants and dyads)

| Individuals | | | | |  | Dyads | | | | | | | |
| --- | --- | --- | --- | --- | --- | --- | --- | --- | --- | --- | --- | --- | --- |
| Age | |  | Gender:  N (%) | |  | Average Age | |  | Gender composition: N (%) | |  | Gender composition: N (%) | |
| Max | 32.69 |  | Male | 50 (62.5%) |  | Max | 27.80 |  | FF | 4 (10%) |  | Same | 18 (45%) |
| Mean | 22.51 |  | Female | 30 (37.5%) |  | Mean | 22.51 |  | MM | 14 (35%) |  |  |  |
| Med | 22.52 |  | Total | 80 (100%) |  | Med | 22.39 |  | FM | 6 (15%) |  | Different | 22 (55%) |
| SD | 3.08 |  |  |  |  | SD | 2.49 |  | MF | 16 (40%) |  |  |  |
| Min | 17.85 |  |  |  |  | Min | 18.28 |  | Total | 40 (100%) |  | Total | 40 (100%) |
| Note: F = Female; M = Male | | | | | | | | | | | | | |

## Supplementary material 2. Instructions given to the participants

| 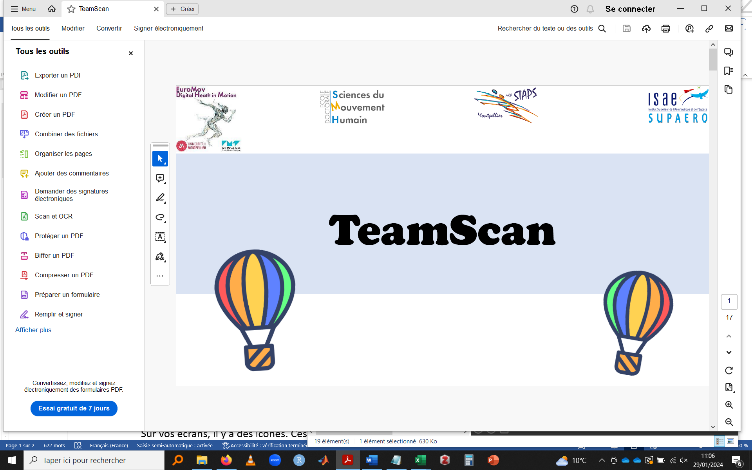 | You participate in the TeamScan experiment; "Team" because you are a team; "Scan" because your brain activity is recorded. |
| --- | --- |
| **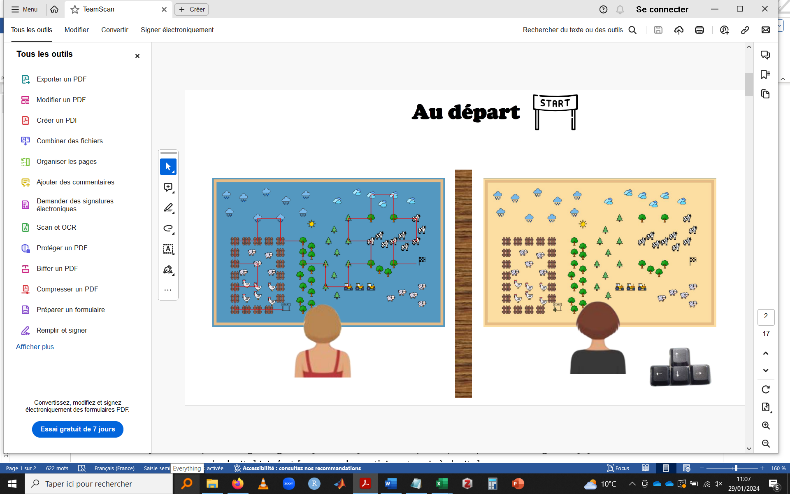** | **Here's the starting point.**  [Point to the character on the left] here is [name Guide]. [Point to the character on the right] here is [name the Drawer].  [Point to the left screen] here is the screen of [name Guide]. [Point to the right screen] here is the screen of [name Drawer].  Before the task begins, I'm going to set up a curtain for you, so that you don't see each other's screens.  On your screens, there are icons. These icons are strictly identical and placed in the same place on both screens; It's the same wallpaper, and it will remain the same throughout the task.  On the [name Guide] screen, there is a path, which goes from a "Start" point, to a "Finish" flag. This path is not present on the screen of [name Designer]. |
| 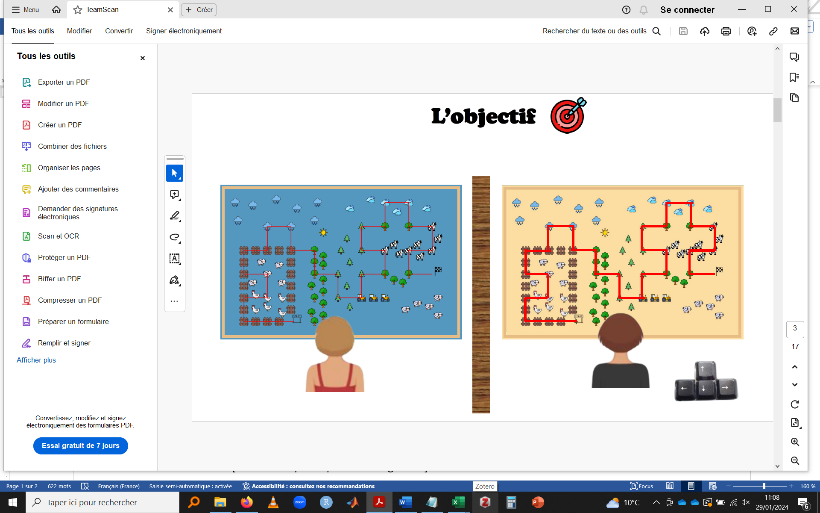 | **Your Goal**  I'm going to ask you to work together, so that at the end of the task there is exactly the same path on the [name Drawer] screen as the one that is already present on the [name Guide] screen.  To do this, [name the Drawer] you will be able to use the arrows on your keyboard to orient a cursor in the shape of a hot air balloon. The track will come out of the hot air balloon basket. |
| 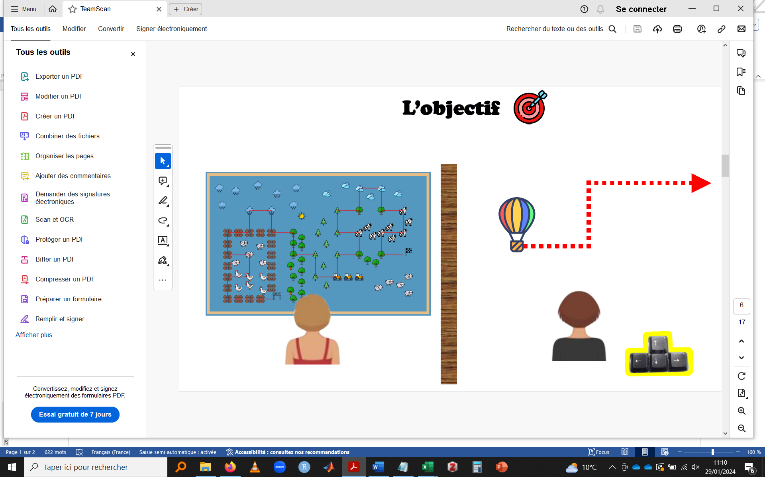 | **How to draw the path?**  At the beginning of the task, the cursor is placed on the "Start" point. As soon as you press an arrow on the keyboard, the balloon will move in a straight directeion, and at a constant speed. In other words, you won't be able to speed up or slow down the cursor. All you can do is make it change direction on the instructions of [name Guide]. |
| **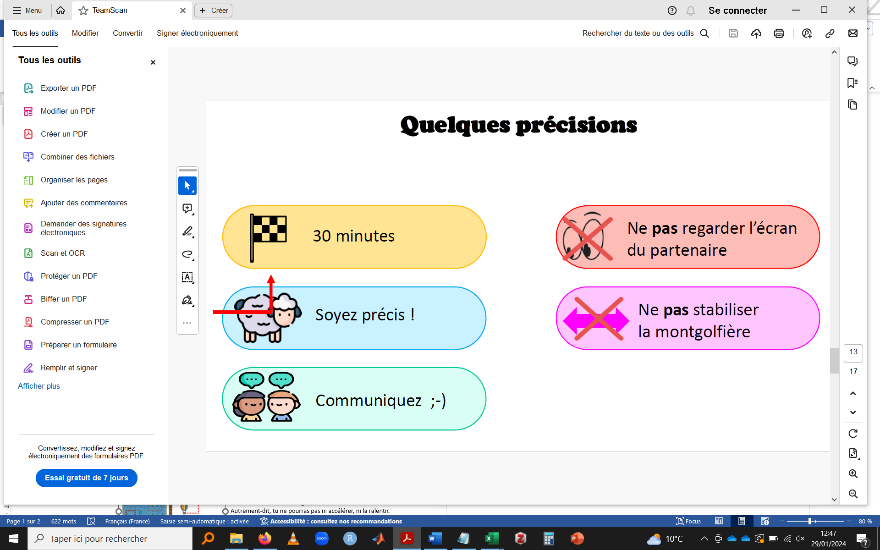** | **A few details**:  The task will take 30 minutes to complete. After 30 minutes, the task stops. In 30 minutes, you won't have time to reach the "Finish" flag.  So your goal is not to reach the Finish flag; it's also not about getting to the Finish flag as fast as possible. Your only goal is to be precise, in other words, to create a path identical to the one already present on the [name Guide] screen.  What does precise mean? For example, if a bend is made on the sheep's ear, the bend should be made on the sheep's ear, not on its back or tail.  During this task, you will communicate, exchange information, feel free to communicate spontaneously with the vocabulary that suits you.  You will not be allowed to look at your partner's screen.  As far as you are concerned [name the Drawer], you will not have the right to "stabilize" the hot air balloon. If you press the opposite arrows, up-down or right-left very quickly, it will prevent the balloon from moving forward, and you are not allowed to do that. Always let the balloon move forward. |
| **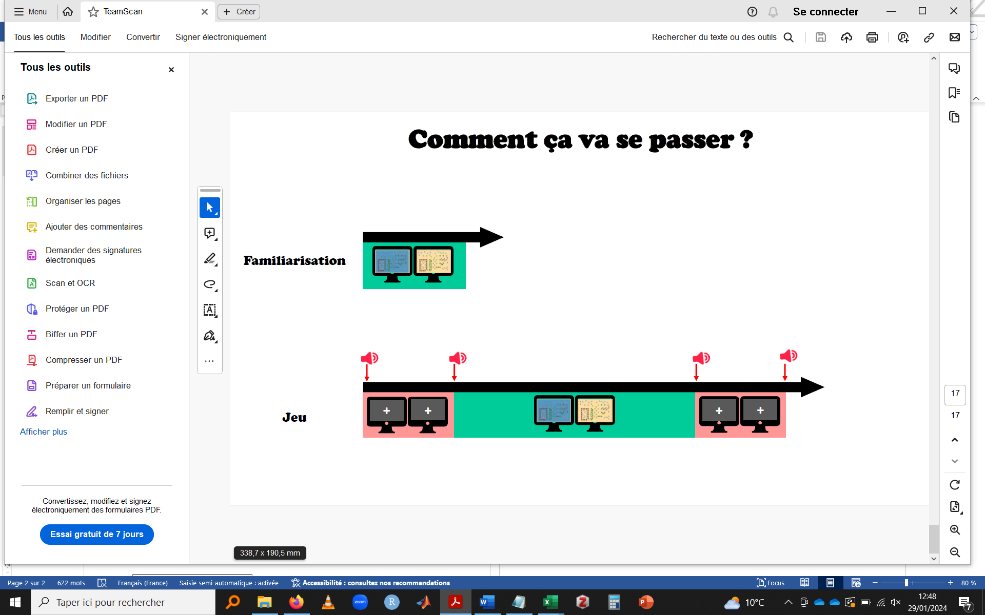** | **How will it work?**  You will complete a 2-minute familiarization task. At the end of it, if you have any questions, I will be able to answer them.  Then you will do the 30-minute task. Before and after this task, you will take a 30-minute rest time. You will see on your screen a white cross on a black background, this is the signal to close your eyes, not to speak, and not to move. I'll let you know when you can open your eyes again. |

## Supplementary material 3. Flow chart of data selection


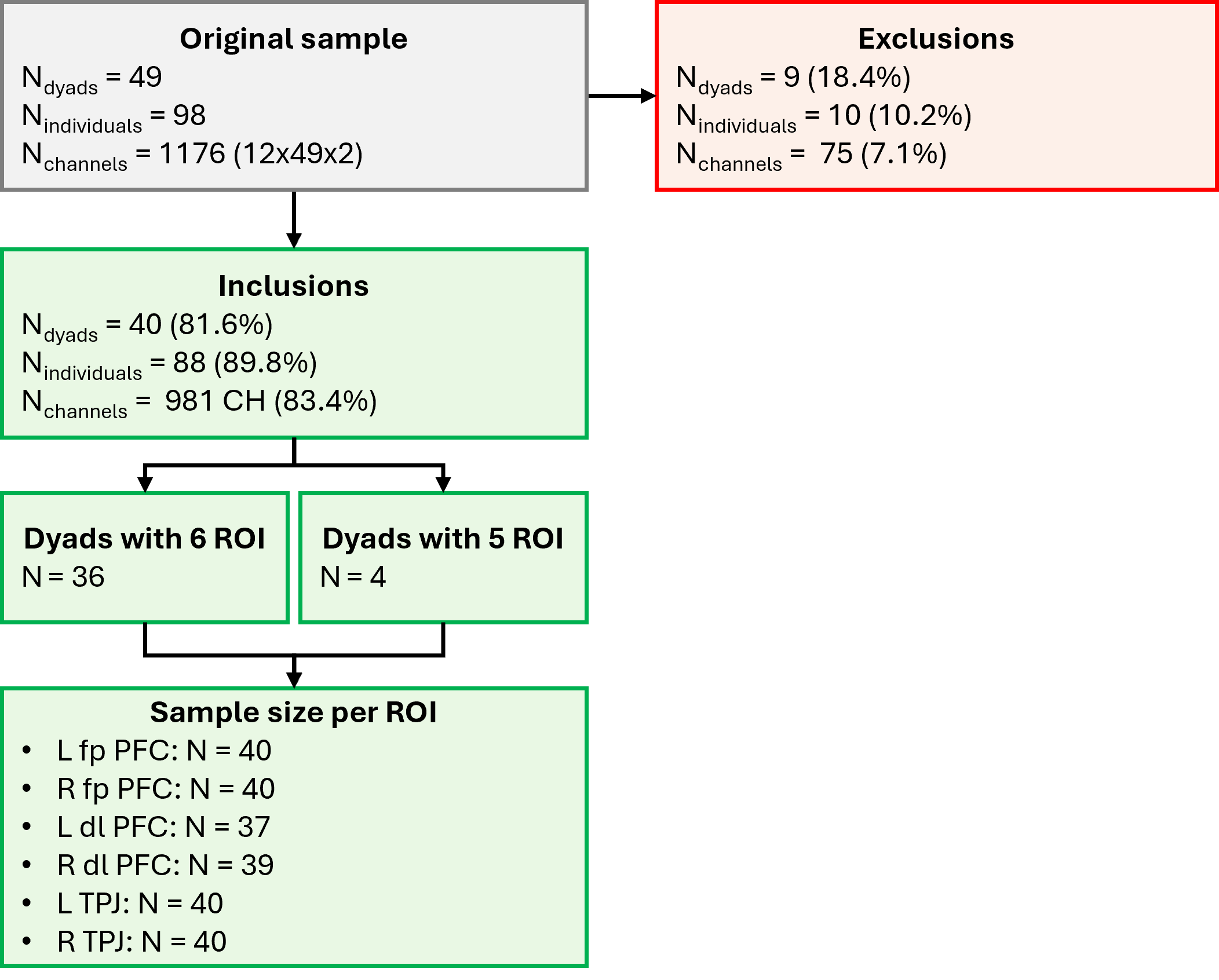


## Supplementary material 4. Visualization of the prediction for the linear mixed models

**
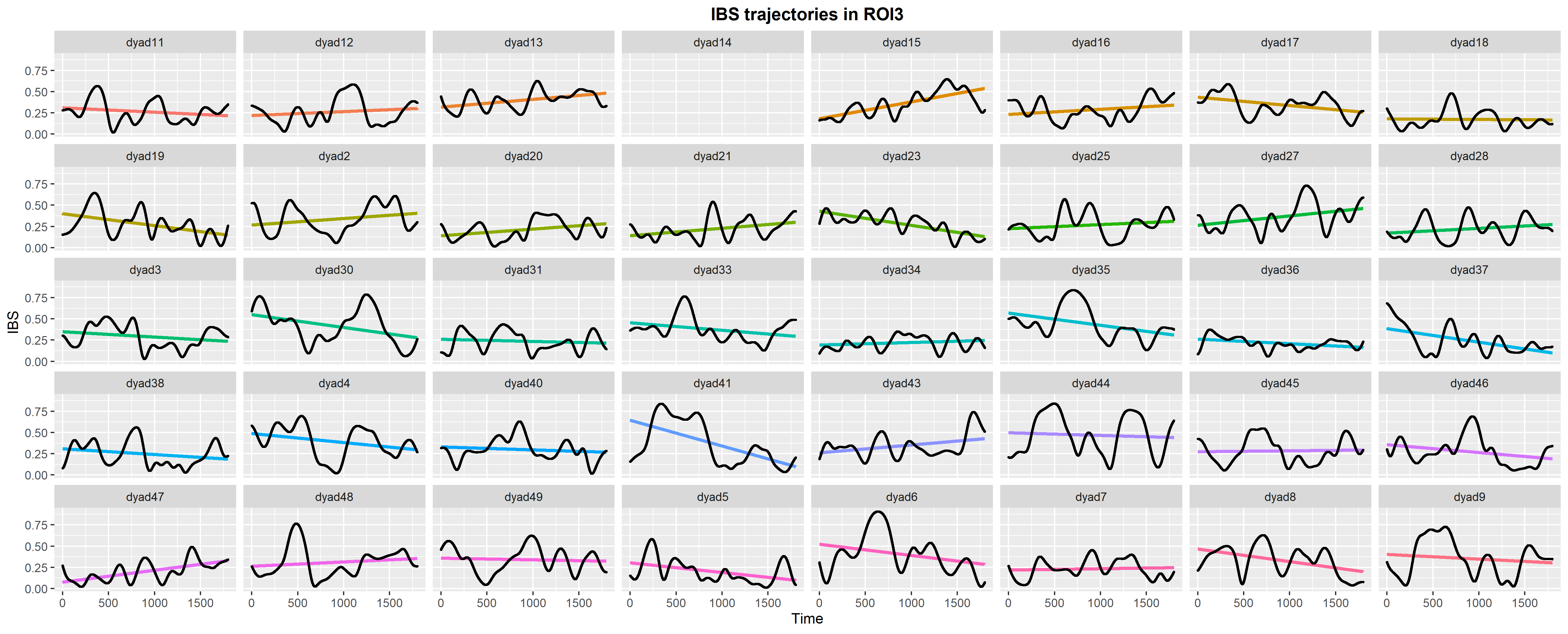
**

***Figure SM-4.1:*** *Prediction for the linear mixed models (Models 2) in ROI 3 (Left dl PFC).*

**
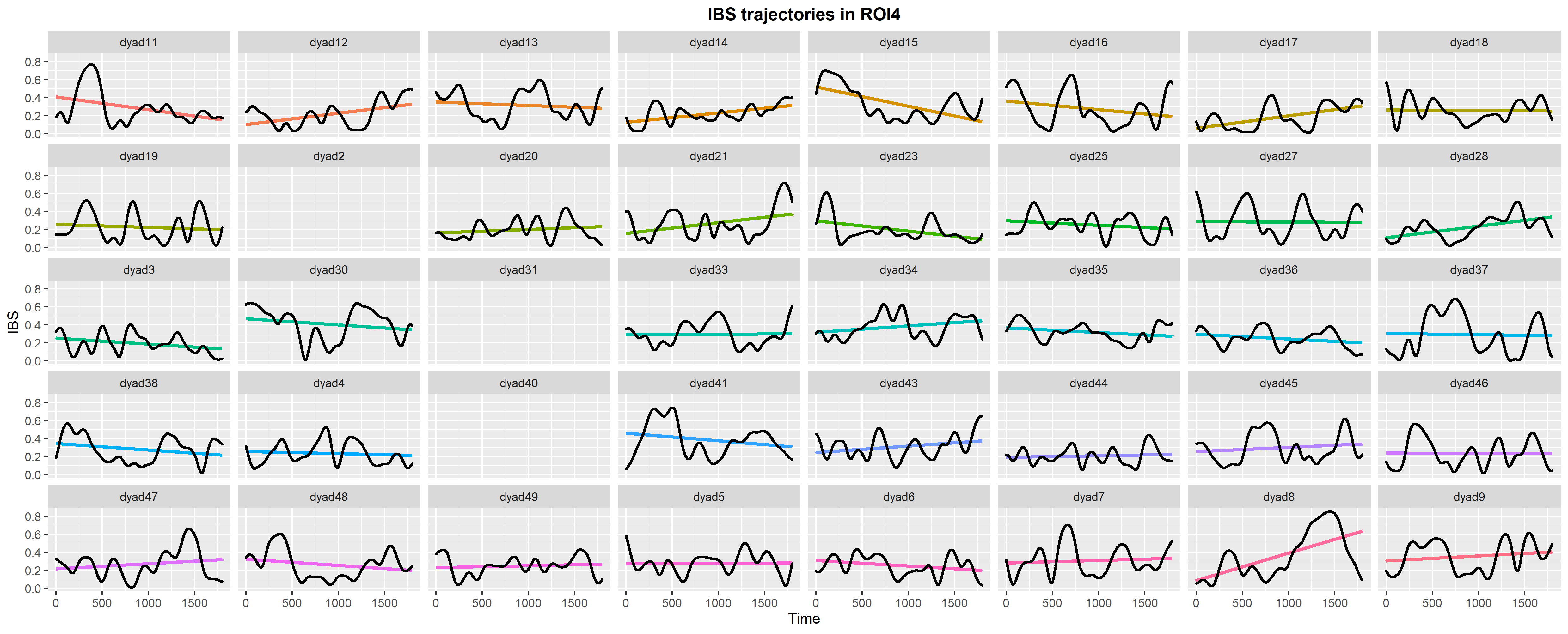
**

***Figure SM-4.2:*** *Prediction for the linear mixed models (Models 2) in ROI 4 (Right dl PFC).*

**
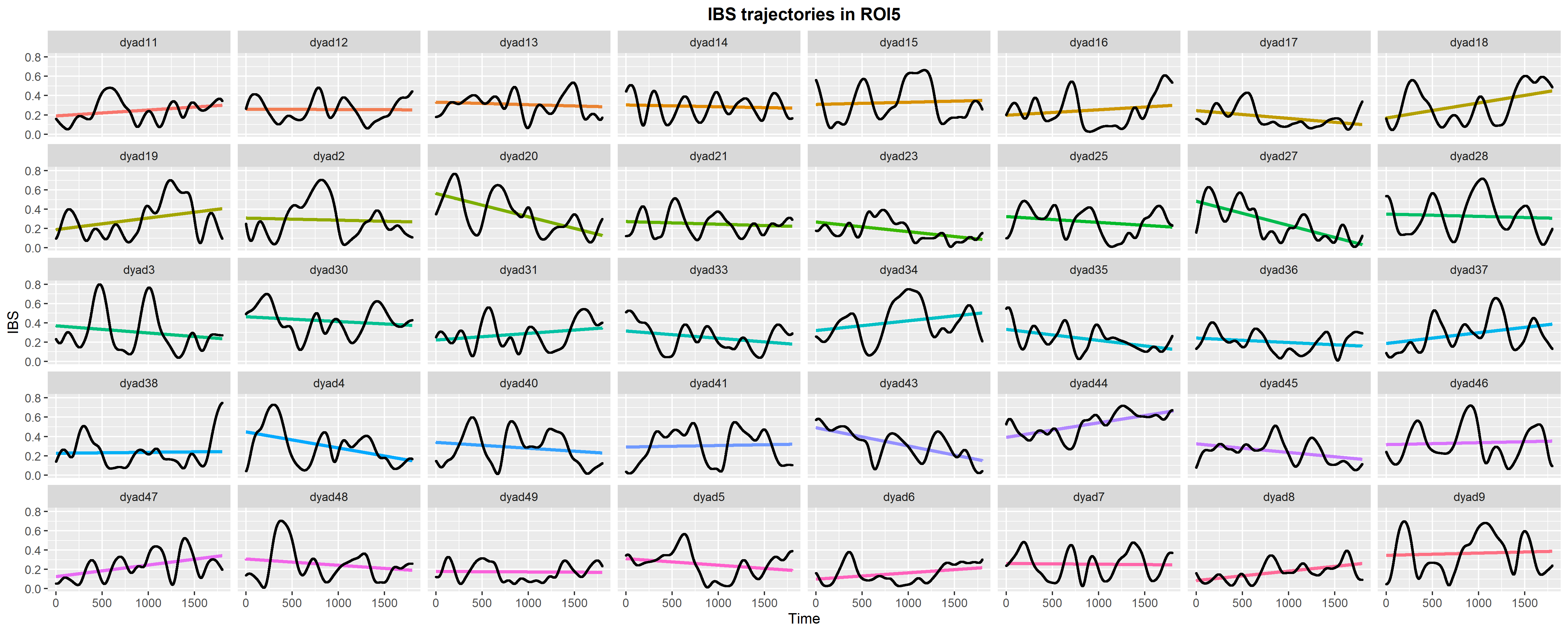
*Figure SM-4.3:*** *Prediction for the linear mixed models (Models 2) in ROI 5 (Left TPJ).*

**
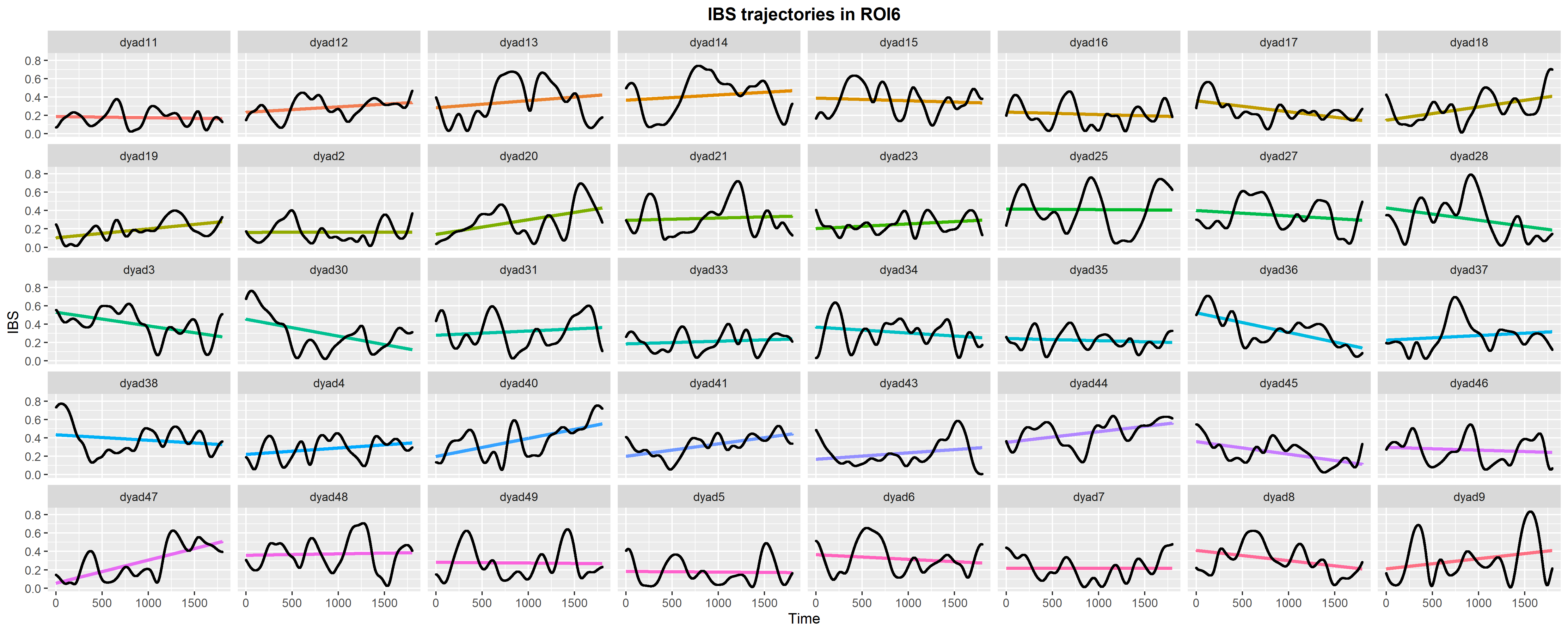
**

***Figure SM-4.4:*** *Prediction for the linear mixed models (Models 2) in ROI 6 (Right TPJ).*

## Supplementary material 5. Relationship between IBS trajectories, team personality and performance

**Table SM-5:** Results of statistical tests for effect of personality on IBS slopes; and for effect of IBS slopes on Performance (Benjamini-Yekutieli FDR corrected). In bold, significant tests.

| Indep Var | Dep Var | df1 | df2 | F | R² | pModel | Beta | StdError | t | pPerso | p_adjBY |
| --- | --- | --- | --- | --- | --- | --- | --- | --- | --- | --- | --- |
| A | Left dl PFC | 1 | 37 | 3.24 | 0.08 | 0.08 | -9.68 x10-5 | 5.38 x10-5 | -1.80 | 0.08 | 1 |
| A | Right dl PFC | 1 | 35 | 0.10 | 0.00 | 0.75 | -1.57 x10-5 | 4.99 x10-5 | -0.31 | 0.75 | 1 |
| A | Left TPJ | 1 | 38 | 0.70 | 0.02 | 0.41 | 4.14 x10-5 | 4.94 x10-5 | 0.84 | 0.41 | 1 |
| A | Righ TPJ | 1 | 38 | 0.32 | 0.01 | 0.57 | 2.98 x10-5 | 5.24 x10-5 | 0.57 | 0.57 | 1 |
|  |  |  |  |  |  |  |  |  |  |  |  |
| E | Left dl PFC | 1 | 37 | 2.05 | 0.05 | 0.16 | 4.78 x10-5 | 3.34 x10-5 | 1.43 | 0.16 | 1 |
| E | Right dl PFC | 1 | 35 | 0.95 | 0.03 | 0.34 | 3.17 x10-5 | 3.26 x10-5 | 0.97 | 0.34 | 1 |
| E | Left TPJ | 1 | 38 | 0.51 | 0.01 | 0.48 | 2.29 x10-5 | 3.20 x10-5 | 0.72 | 0.48 | 1 |
| E | Righ TPJ | 1 | 38 | 0.41 | 0.01 | 0.53 | 2.17 x10-5 | 3.38 x10-5 | 0.64 | 0.53 | 1 |
|  |  |  |  |  |  |  |  |  |  |  |  |
| C | Left dl PFC | 1 | 37 | 0.81 | 0.02 | 0.37 | 3.57 x10-5 | 3.96 x10-5 | 0.90 | 0.37 | 1 |
| C | Right dl PFC | 1 | 35 | 0.14 | 0.00 | 0.71 | -1.39 x10-5 | 3.72 x10-5 | -0.37 | 0.71 | 1 |
| C | Left TPJ | 1 | 38 | 0.01 | 0.00 | 0.92 | 3.67 x10-6 | 3.73 x10-5 | 0.10 | 0.92 | 1 |
| C | Righ TPJ | 1 | 38 | 0.38 | 0.01 | 0.54 | -2.41 x10-5 | 3.91 x10-5 | -0.61 | 0.54 | 1 |
|  |  |  |  |  |  |  |  |  |  |  |  |
| N | Left dl PFC | 1 | 37 | 1.20 | 0.03 | 0.28 | -3.32 x10-5 | 3.04 x10-5 | -1.09 | 0.28 | 1 |
| N | Right dl PFC | 1 | 35 | 0.06 | 0.00 | 0.81 | -7.04 x10-6 | 2.96 x10-5 | -0.24 | 0.81 | 1 |
| N | Left TPJ | 1 | 38 | 0.04 | 0.00 | 0.85 | -5.46 x10-6 | 2.89 x10-5 | -0.19 | 0.85 | 1 |
| N | Righ TPJ | 1 | 38 | 0.13 | 0.00 | 0.72 | 1.08 x10-5 | 3.05 x10-5 | 0.35 | 0.72 | 1 |
|  |  |  |  |  |  |  |  |  |  |  |  |
| O | Left dl PFC | 1 | 37 | 0.81 | 0.02 | 0.37 | -3.37 x10-5 | 3.74 x10-5 | -0.90 | 0.37 | 1 |
| O | Right dl PFC | 1 | 35 | 0.45 | 0.01 | 0.51 | 2.26 x10-5 | 3.37 x10-5 | 0.67 | 0.51 | 1 |
| **O** | **Left TPJ** | **1** | **38** | **5.55** | **0.13** | **0.02** | **7.56 x10-5** | **3.21 x10-5** | **2.36** | **0.02** | **1** |
| O | Righ TPJ | 1 | 38 | 0.00 | 0.00 | 0.98 | 7.99 x10-7 | 3.63 x10-5 | 0.02 | 0.98 | 1 |
|  |  |  |  |  |  |  |  |  |  |  |  |
| Left dl PFC | Perf | 1 | 37 | 0.42 | 0.01 | 0.52 | -1926636 | 2974737 | -0.65 | 0.52 | 1 |
| Right dl PFC | Perf | 1 | 35 | 0.76 | 0.02 | 0.39 | 2968591 | 3402451 | 0.87 | 0.39 | 1 |
| Left TPJ | Perf | 1 | 38 | 2.24 | 0.06 | 0.14 | 4516914 | 3016150 | 1.50 | 0.14 | 1 |
| Righ TPJ | Perf | 1 | 38 | 0.92 | 0.02 | 0.34 | 2783203 | 2908639 | 0.96 | 0.34 | 1 |
|  |  |  |  |  |  |  |  |  |  |  |  |
| Notes: A = Agreeableness; E = Extroversion; C = Conscientiousness; N = Neuroticism; O = Openness to experiences; Perf = Performance; fp = frontopolar; dl = dorsolateral; PFC = prefrontal cortex; TPJ = temporo-parietal junction; Indep Var = Independent variable ; Dep Var = Dependant Variable ; df = degree of freedom; pModel = p value of the model; pIV = p value of the Indépendant Variable ; p_adj_BY = p value of the Independant Variable corrected with the Benjamini-Yekutieli correction. | | | | | | | | | | | |

## Supplementary material 6. Analyses of deoxyhaemoglobin (HHb)

### 6.1 Results of the control analyses for (HHb)

**Table SM-6.1:** Results of the control analyses (t-tests) for the frequency band of interest.

| ROI | IBS in Real dyads | IBS in Permuted dyads | t | p | Cohen's d |  |
| --- | --- | --- | --- | --- | --- | --- |
|  | Mean (SD) | Mean (SD) |  |  |  |  |
| Left fp PFC | 0.27 (0.06) | 0.27 (0.06) | 0.41 | 0.34 | 0.07 |  |
| Right fp PFC | 0.27 (0.08) | 0.27 (0.07) | 0.26 | 0.40 | 0.04 |  |
| Left dl PFC | 0.26 (0.07) | 0.26 (0.07) | 0.36 | 0.36 | 0.06 |  |
| **Right dl PFC** | **0.28 (0.05)** | **0.24 (0.09)** | **2.82** | **0.00** | **0.47** |  |
| **Left TPJ** | **0.29 (0.05)** | **0.27 (0.05)** | **2.10** | **0.02** | **0.34** |  |
| Right TPJ | 0.27 (0.06) | 0.26 (0.05) | 1.29 | 0.10 | 0.21 |  |
| *Notes:* fp = frontopolar; dl = dorsolateral; PFC = prefrontal cortex; TPJ = temporoparietal junction; SD = standard deviation | | | | | | |

**Results:** Using HHb data, only two out of the six ROIs under investigation – the right dlPFC and the left TPJ – showed higher IBS for Real dyads compared to Permuted dyads.

**Conclusion:** Subsequent analyses for HHb will therefore focus on two ROIs : right dlPFC and left TPJ.

###
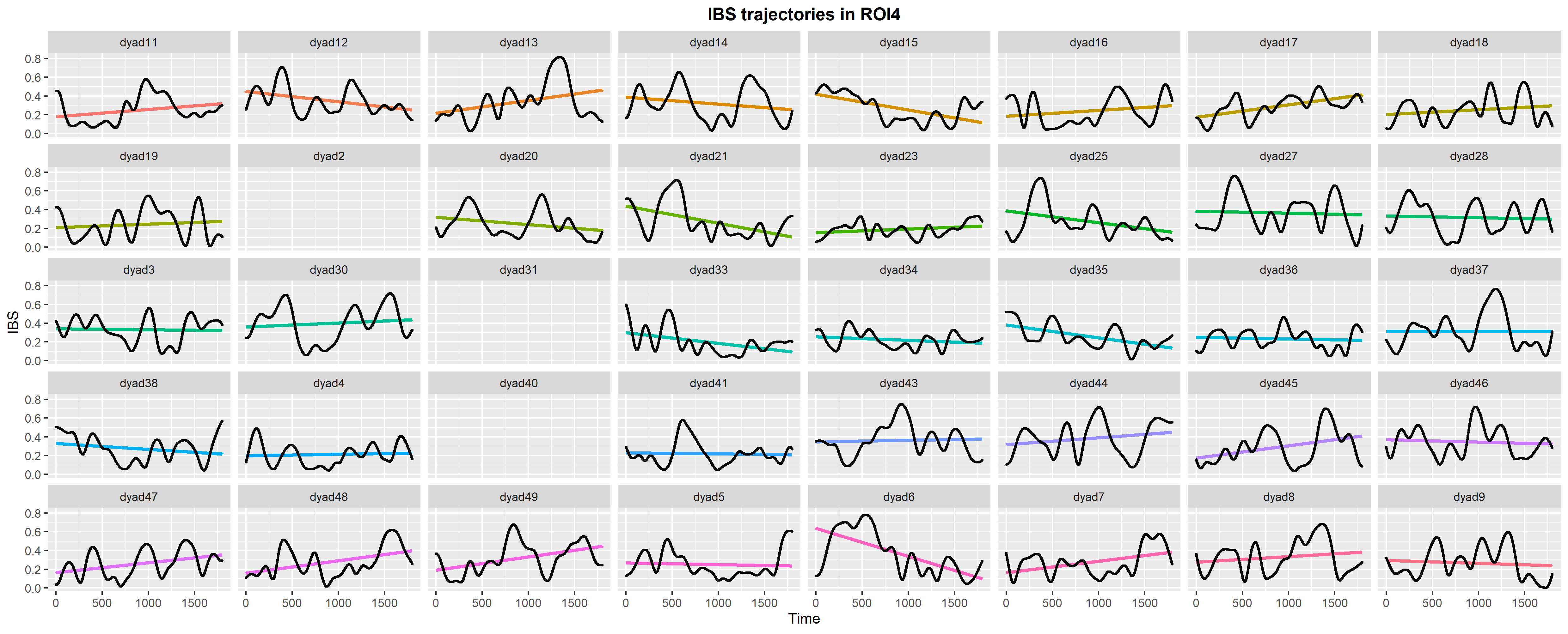
6.2 Visualization of the prediction for the Linear Mixed Models (HHb)

***Figure SM-6.1:*** *Prediction for the linear mixed models (Models 2) in ROI 4 (Right dl PFC).*

*
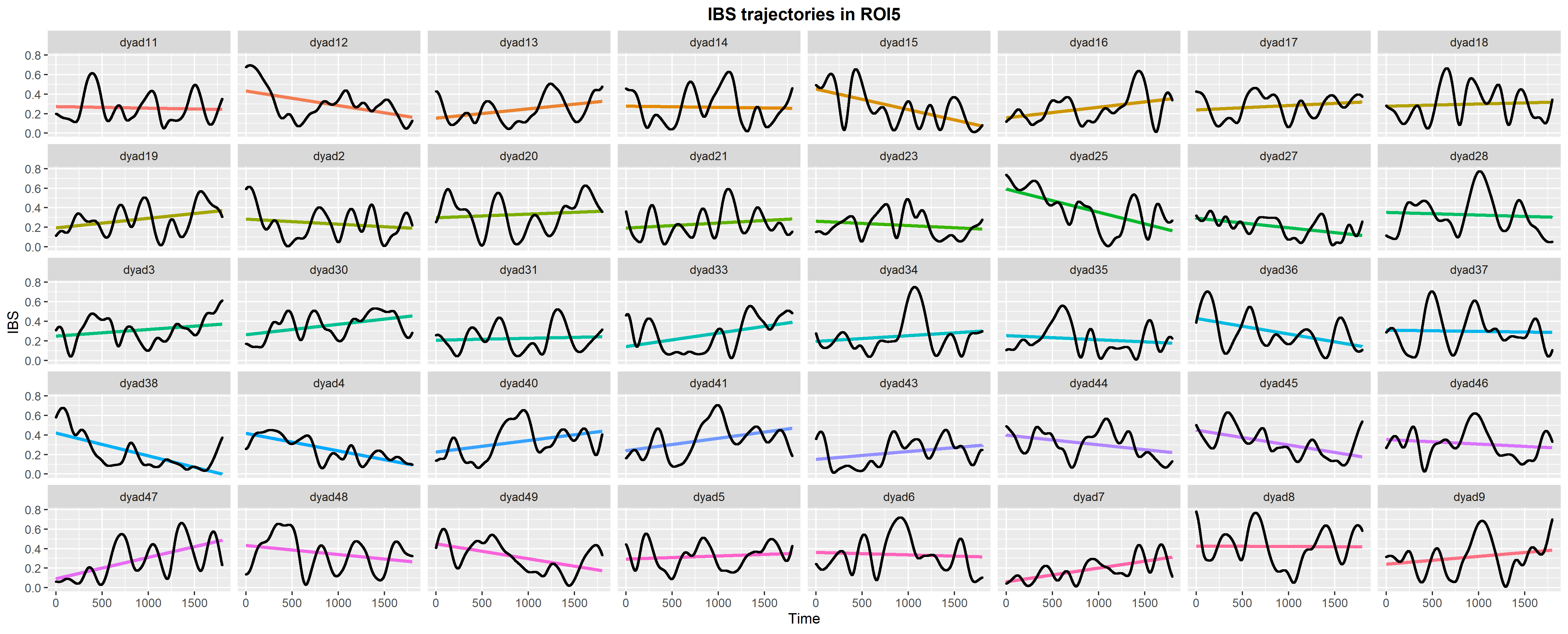
****Figure SM-6.1:*** *Prediction for the linear mixed models (Models 2) in ROI 5 (Left TPJ).*

### 6.2. Results of the Linear Mixed Models (HHb)

#### 6.2.1. Effect of time

**Table SM-6.2:** Results of linear mixed models with random effect of time in the two ROIs (Full Models) for HHb

|  |  |  | Intercept | | |  | Slope | | |  |
| --- | --- | --- | --- | --- | --- | --- | --- | --- | --- | --- |
| ROI | N |  | Value | t | p |  | Value | t | p |  |
| Right dl PFC | 37 |  | 0.29 | 11.61 | 6.59 x10-9 |  | -4.53 x10-6 | -0.07 | 0.94 |  |
| Left TPJ | 40 |  | 0.29 | 23.64 | 1.11 x10-44 |  | -1.00 x10-5 | -0.69 | 0.49 |  |
| *Notes:* fp = frontopolar; dl = dorsolateral; PFC = prefrontal cortex; TPJ = temporoparietal junction; SD = standard deviation | | | | | | | | | |  |
|  |  |  |  |  |  |  |  |  |  |  |

**Results:** In the two ROIs under investigation, the intercept values are significantly different from 0 (p < 0.001). However, the slope values do not significantly differ from 0 (p > 0.94).

**Interpretation:** In none of the two ROIs does the trajectory of IBS show a trend of increase or decrease within the studied sample.

#### 6.2.2. Inter-team differences

**Table SM-6.3:** Results of the model comparisons in the two ROIs (Model 2 vs. Model 1) for HHb

|  | Model 1 (No random slope) | | |  | Model 2 (Random slope) | | |  | ANOVA | | |  |
| --- | --- | --- | --- | --- | --- | --- | --- | --- | --- | --- | --- | --- |
| ROI | AIC | BIC | logLik |  | AIC | BIC | logLik |  | Chi² | df | p |  |
| Right dl PFC | -92432 | -92394 | 46220 |  | -104826 | -104768 | 52419 |  | 12398 | 2 | <0.001 |  |
| Left TPJ | -103643 | -103605 | 51826 |  | -121200 | -121142 | 60606 |  | 17561 | 2 | <0.001 |  |
| *Notes:* AIC = Akaike Information Criterion; BIC = Bayesian Information Criterion; logLik = log-likelihood; df = Degree of Freedom. | | | | | | | | | | | |  |
|  |  |  |  |  |  |  |  |  |  |  |  |  |

**Results:** In the two ROIs, the AIC and BIC values are lower in Model 1 (without a random slope) compared to Model 2 (with a random slope), and the comparison between the two models is significant (p < 0.001).

**Interpretation:** There are significant inter-team differences in IBS trajectories across the two ROIs.

### 6.3. Relationship between IBS trajectories. team personality and performance (HHb)

**Table SM-6.4:** Results of statistical tests for effect of personality on IBS slopes in HHb; and for effect of IBS slopes in HHb on Performance (Benjamini-Yekutieli FDR corrected).

| Indep Var | Dep Var | df1 | df2 | F | R² | P  Model | Beta | StdError | t | pPerso | p_adjBY |
| --- | --- | --- | --- | --- | --- | --- | --- | --- | --- | --- | --- |
| A | Right dl PFC | 1 | 35 | 0.66 | 0.02 | 0.42 | -4.34 x10-5 | 5.36 x10-5 | -0.81 | 0.42 | 1.00 |
| A | Left TPJ | 1 | 38 | 1.12 | 0.03 | 0.30 | 6.06 x10-5 | 5.73 x10-5 | 1.06 | 0.30 | 1.00 |
| E | Right dl PFC | 1 | 35 | 2.70 | 0.07 | 0.11 | -5.66 x10-5 | 3.45 x10-5 | -1.64 | 0.11 | 1.00 |
| E | Left TPJ | 1 | 38 | 0.03 | 0.00 | 0.87 | -6.12 x10-6 | 3.75 x10-5 | -0.16 | 0.87 | 1.00 |
| C | Right dl PFC | 1 | 35 | 0.11 | 0.00 | 0.74 | -1.36 x10-5 | 4.03 x10-5 | -0.34 | 0.74 | 1.00 |
| C | Left TPJ | 1 | 38 | 9.85 | 0.21 | 0.00 | -1.22 x10-4 | 3.87 x10-5 | -3.14 | 0.00 | 0.10 |
| N | Right dl PFC | 1 | 35 | 0.05 | 0.00 | 0.82 | 7.37 x10-6 | 3.20 x10-5 | 0.23 | 0.82 | 1.00 |
| N | Left TPJ | 1 | 38 | 0.63 | 0.02 | 0.43 | 2.66 x10-5 | 3.34 x10-5 | 0.80 | 0.43 | 1.00 |
| O | Right dl PFC | 1 | 35 | 0.00 | 0.00 | 0.98 | -8.80 x10-7 | 3.67 x10-5 | -0.02 | 0.98 | 1.00 |
| O | Left TPJ | 1 | 38 | 0.59 | 0.02 | 0.45 | 3.06 x10-5 | 3.97 x10-5 | 0.77 | 0.45 | 1.00 |
| Right dl PFC | Perf | 1 | 35 | 1.87 | 0.05 | 0.18 | 4235083 | 3095647 | 1.37 | 0.18 | 0.27 |
| Left TPJ | Perf | 1 | 38 | 2.84 | 0.07 | 0.10 | 4334218 | 2570785 | 1.69 | 0.10 | 0.27 |
| Notes: A = Agreeableness; E = Extroversion; C = Conscientiousness; N = Neuroticism; O = Openness to experiences; Perf = Performance; fp = frontopolar; dl = dorsolateral; PFC = prefrontal cortex; TPJ = temporo-parietal junction; Indep Var = Independent variable ; Dep Var = Dependant Variable ; df = degree of freedom; pModel = p value of the model; pIV = p value of the Indépendant Variable ; p_adj_BY = p value of the Independant Variable corrected with the Benjamini-Yekutieli correction. | | | | | | | | | | | |

**Results:** After FDR correction, team personality composition does not predict IBS trajectories in HHb data in any of the two ROIs. Similarly, none of the two IBS trajectories predict performance.

**Interpretation:** IBS trajectories in HHb data are not related to team personality composition or performance.

## Supplementary material 7. Complementary analyses: does average IBS predict team performance in O_2_Hb data?

### 7.1. Rationale

As we found that IBS slopes did not predict team performance, we explored whether the overall level of IBS could do so instead.

### 7.2. Statistical analysis

We conducted linear regressions with team performance as the dependent variable and the average level of IBS as the independent variable.

### 7.3. Results

Results showed that in none of the four ROIs under investigation in O_2_Hb data, the average level of IBS significantly predict team performance (see ***Table SM-7).***

**Table SM-7:** Results of the linear regression testing if average level of IBS predict team performance

| ROI | Fdf1 | Fdf2 | Fvalue | R2 | pModel | Beta | StdError | tValue | pValue | p_adj |
| --- | --- | --- | --- | --- | --- | --- | --- | --- | --- | --- |
| Left dl PFC | 1 | 37 | 0.00 | 0.00 | 0.97 | 133.78 | 4172.83 | 0.03 | 0.97 | 1 |
| Right dl PFC | 1 | 35 | 1.73 | 0.05 | 0.20 | 6883.47 | 5240.60 | 1.31 | 0.20 | 1 |
| Left TPJ | 1 | 38 | 0.47 | 0.01 | 0.50 | 2838.79 | 4157.13 | 0.68 | 0.50 | 1 |
| Righ TPJ | 1 | 38 | 1.81 | 0.05 | 0.19 | 5624.12 | 4178.20 | 1.35 | 0.19 | 1 |
| Notes: fp = frontopolar; dl = dorsolateral; PFC = prefrontal cortex; TPJ = temporoparietal junction; SD = standard deviation | | | | | | | | | | |

### 7.4. Discussion

This result does not align with previous studies reporting that the average level of IBS can predict performance during teamwork (Réveillé et al., 2024).

## Supplementary material 8. Complementary analyses: patterns of IBS trajectories across the four ROIs (O_2_Hb)

### 8.1. Rationale

As we found that the main effect of time was homogeneous across the four ROIs (i.e. no significant overall effect of time), we sought to further investigate potential heterogeneity at the level of individual ROIs. Specifically. we aimed to determine whether teams exhibited similar patterns of IBS change across different ROIs. or whether certain regions followed distinct trajectories.

### 8.2. Statistical analysis

We tested for differences in IBS slopes across the four ROIs using a repeated measures ANOVA. with ROIs as a within-subject factor. This allowed us to assess whether the observed variations in IBS trajectories were consistent across brain regions within teams or whether specific ROIs demonstrated divergent trends.

### 8.3. Results

Results showed no significant difference in IBS slopes across ROIs (F(3,105) = 0.777; p = 0.51).

A correlogram showing correlations among IBS slopes in the four ROIs is available in ***Figure SM-8.***

### 8.4. Discussion

We found that IBS evolves similarly across cortical areas involved in social cognition (i.e. PFC and TPJ). This finding should be treated as preliminary and requires further replication; however. if confirmed. it could provide a deeper understanding of the temporal dynamics of brain networks. shedding light on how cognitive processes unfold during a social interaction such as teamwork.


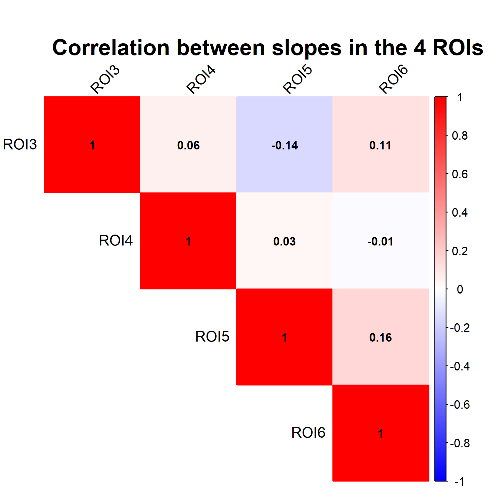


***Figure SM-8:*** *Correlogram of IBS slopes across the four brain regions*

## Supplementary material 9. Complementary analyses: classification of the teams according to their IBS trajectories

### 9.1. Rational

To further investigate the inter-team differences in IBS slopes. while accounting for the IBS trajectories in the four ROIs. we also conducted data driven analyses (Li et al.. 2021; Wang et al.. 2019).

### 9.2. Analyses

K-mean clustering was computed on IBS slopes values of the 36 dyads with data available in the four ROIs. The optimal number of clusters was searched using the elbow criterion, based on the intra-cluster distances (Within Sum of Squares. WSS).

### 9.3. Detailed methods of clustering

- Dataset: IBS slope per dyads in the four ROIs for 36 dyads with no missing data. N_observations_ = 36; N_dimensions_ = 4.
- Software: *K-means* clustering as conducted using Rstudio using the {cluster}package.
- Step 1: Identification of the optimal number of clusters using the *K-means* clustering algorithm with « wss » function.

- The k-mean algorithm was ran using different number of clusters. from k = 1 to k =10.

- The nstart parameter was set at 25. This mean that for each value of k tested. the algorithm was running 25 times with different initial cluster assignments. The solution with the lowest WSS was retained. to enhance reliability of the results. WSS represents the average across clusters of the squared distances between each data point and the cluster's centroid. was calculated.

- The total within-cluster sum of squares (WSS) was extracted. The lower WSS value. the more compact the cluster.

- The elbow methods were applied across the range of k tested. WSS values are plotted against k. the "elbow point" on the curve - where the rate of decrease in WSS lower significantly - suggests the appropriate number of clusters.

- Step 2: application of the K-mean algorithm with the optimal number of clusters.
- Step 3: extraction of models’ indicators:

- Silhouette score of the model

- Centroid value of each cluster for each ROI

- ROI importance: by calculating the change of IBS slopes across clusters. This represents the magnitude of change in the centroids of each variable across clusters. helping to evaluate which variables vary the most between clusters and. consequently. which are the most important for differentiating the clusters.

### 9.4. Detailed results of clustering

The elbow method failed to reveal a clear inflection point, making it impossible to determine a justifiable number of clusters (see ***Figure 9-1***). Therefore, no robust clustering solution could be established, and we chose not to proceed with the clustering analysis.


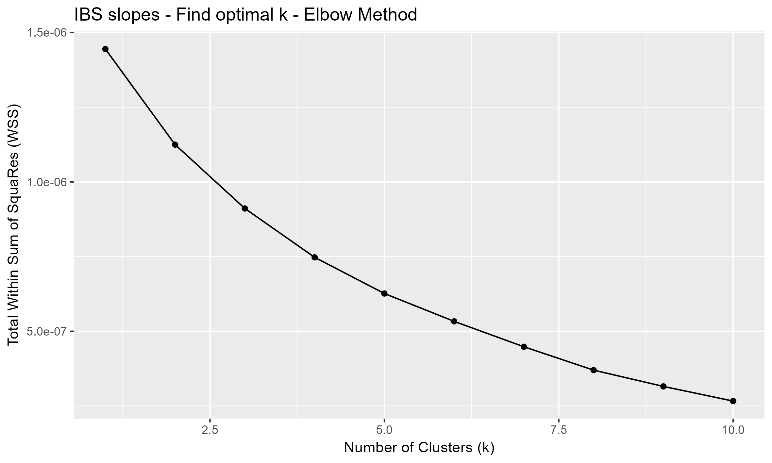
***Figure SM-9.1:*** *figure of the elbow method.*
